# (*E*)-2,2′-[3-(2-Nitro­phen­yl)prop-2-ene-1,1-di­yl]bis­(3-hy­droxy­cyclo­hex-2-en-1-one)

**DOI:** 10.1107/S1600536811054730

**Published:** 2011-12-23

**Authors:** Joo Hwan Cha, Young Hee Kim, Jae Kyun Lee, Yong Seo Cho

**Affiliations:** a Advanced Analysis Center, Korea Institute of Science & Technology, Hwarangro 14-gil, Seongbuk-gu, Seoul 136-791, Republic of Korea; b Center for Neuro-Medicine, Korea Institute of Science & Technology, Hwarangro 14-gil, Seongbuk-gu, Seoul 136-791, Republic of Korea

## Abstract

In the title compound, C_21_H_21_NO_6_, each of the cyclo­hexenone rings adopts a half-chair conformation. Each of the pairs of hy­droxy and carbonyl O atoms are oriented to allow for the formation of intra­molecular O—H⋯O hydrogen bonds, which are typical of xanthene derivatives.

## Related literature

For the biological activity xanthenes and their derivatives and for related structures, see: Lee *et al.* (2011 ▶).
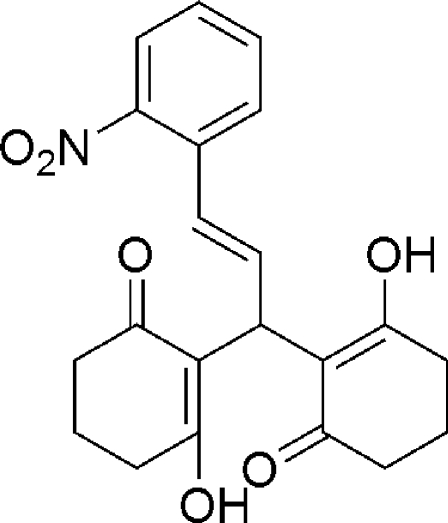


## Experimental

### 

#### Crystal data

C_21_H_21_NO_6_
*M* *_r_* = 383.40
Monoclinic, 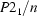

*a* = 8.0785 (7) Å
*b* = 8.7261 (6) Å
*c* = 26.2616 (17) Å
β = 90.829 (3)°
*V* = 1851.1 (3) Å^3^
*Z* = 4
Mo *K*α radiation
μ = 0.10 mm^−1^
*T* = 296 K
0.30 × 0.20 × 0.10 mm

#### Data collection

Rigaku R-AXIS RAPID diffractometer
Absorption correction: multi-scan (*ABSCOR*; Rigaku, 1995 ▶) *T*_min_ = 0.739, *T*_max_ = 0.990
14705 measured reflections
3387 independent reflections
2335 reflections with *F* ^2^ > 2σ(*F* ^2^)
*R*_int_ = 0.028

#### Refinement

*R*[*F* ^2^ > 2σ(*F* ^2^)] = 0.045
*wR*(*F* ^2^) = 0.156
*S* = 1.14
3387 reflections
263 parameters
H atoms treated by a mixture of independent and constrained refinement
Δρ_max_ = 0.31 e Å^−3^
Δρ_min_ = −0.24 e Å^−3^

### 

Data collection: *RAPID-AUTO* (Rigaku, 2006 ▶); cell refinement: *RAPID-AUTO*; data reduction: *RAPID-AUTO*; program(s) used to solve structure: *SHELXS97* (Sheldrick, 2008 ▶); program(s) used to refine structure: *SHELXL97* (Sheldrick, 2008 ▶); molecular graphics: *CrystalStructure* (Rigaku, 2010 ▶); software used to prepare material for publication: *CrystalStructure*.

## Supplementary Material

Crystal structure: contains datablock(s) global, I. DOI: 10.1107/S1600536811054730/ff2047sup1.cif

Structure factors: contains datablock(s) I. DOI: 10.1107/S1600536811054730/ff2047Isup2.hkl

Supplementary material file. DOI: 10.1107/S1600536811054730/ff2047Isup3.cml

Additional supplementary materials:  crystallographic information; 3D view; checkCIF report

## Figures and Tables

**Table 1:** Hydrogen-bond geometry (Å, °)

*D*—H⋯*A*	*D*—H	H⋯*A*	*D*⋯*A*	*D*—H⋯*A*
O2—H2⋯O3	0.82	1.83	2.627 (2)	164
O4—H4⋯O1	0.82	1.78	2.579 (2)	165

**Fig. 1.**
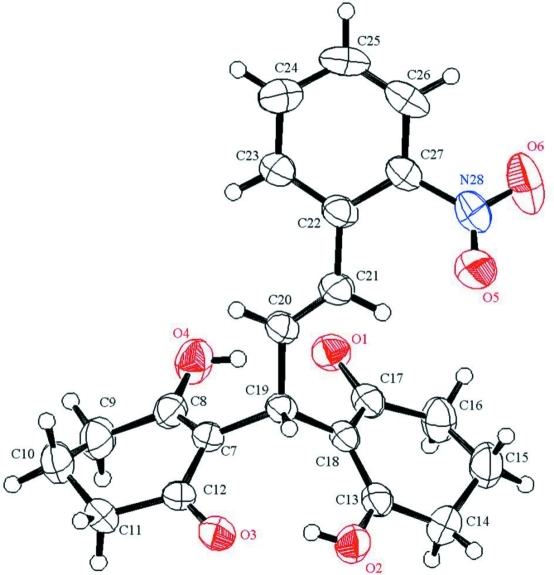
The molecular structure of (I) showing the atomic numbering and 50% probability displacement ellipsoid.

## supplementary crystallographic information

### Comment

As part of our ongoing study of the substituent effect on the solid state structures of xanthene derivatives (Lee *et al.*, 2011). We present here the crystal structure of the title compound (I) (Fig. 1).

In (I) (Fig. 1), the bond lengths and angles are normal and correspond to those observed in related structures Lee *et al.*, 2011). In the title compound, both cyclohexenone rings in (Fig.1) display half-chair conformation. The nitro group is not co-planar with the benzene ring to which it is attached as seen in the O5—N28—C27—C26 torsion angle of 141.7 (2)°. The hydroxy and carbonyl O atoms face each other and are orientated to allow for the formation of two intramolecular O—H···O hydrogen bonds (Table 1) which are typical of xanthene derivatives.

### Experimental

To solution of 1,3-cyclohexanedione (4.61 mmol), 2-nitrocinnamaldehyde (1.84 mmol) and 4Å MS was added catalytic amounts of *L*-proline (0.47 mmol) in under nitrogen atmosphere. After stirring for 2 h, The anhydrous ethyl acetate (0.5 ml) was added to a reaction mixture and the solution was stirred for 12 h. The reaction mixture was filtered through pad of celite to remove MS and concentrated. The residue oil was purified by flash column chromatography to afford product which was recrystallized from ethanol to give yellow crystals suitable for X-ray analysis.

### Refinement

All hydrogen atoms were positioned geometrically and refined using a riding model with C—H = 0.91–0.98 Å and *U*_iso_(H) = 1.2 *U*_eq_(C,*O*). A rotating model was used for OH groups.

### Crystal data

**Table:**

C_21_H_21_NO_6_	*F*(000) = 808.00
*M**_r_* = 383.40	*D*_x_ = 1.376 Mg m^−^^3^
Monoclinic, *P*2_1_/*n*	Mo *K*α radiation, λ = 0.71075 Å
Hall symbol: -P 2yn	Cell parameters from 11386 reflections
*a* = 8.0785 (7) Å	θ = 3.1–27.5°
*b* = 8.7261 (6) Å	µ = 0.10 mm^−^^1^
*c* = 26.2616 (17) Å	*T* = 296 K
β = 90.829 (3)°	Block, colourless
*V* = 1851.1 (3) Å^3^	0.30 × 0.20 × 0.10 mm
*Z* = 4	

### Data collection

**Table:**

Rigaku R-AXIS RAPID diffractometer	2335 reflections with *F*^2^ > 2σ(*F*^2^)
Detector resolution: 10.000 pixels mm^-1^	*R*_int_ = 0.028
ω scans	θ_max_ = 25.4°
Absorption correction: multi-scan (*ABSCOR*; Rigaku, 1995)	*h* = −9→9
*T*_min_ = 0.739, *T*_max_ = 0.990	*k* = −10→9
14705 measured reflections	*l* = −31→31
3387 independent reflections	

### Refinement

**Table:**

Refinement on *F*^2^	Secondary atom site location: difference Fourier map
*R*[*F*^2^ > 2σ(*F*^2^)] = 0.045	Hydrogen site location: inferred from neighbouring sites
*wR*(*F*^2^) = 0.156	H atoms treated by a mixture of independent and constrained refinement
*S* = 1.14	*w* = 1/[σ^2^(*F*_o_^2^) + (0.0927*P*)^2^] where *P* = (*F*_o_^2^ + 2*F*_c_^2^)/3
3387 reflections	(Δ/σ)_max_ = 0.026
263 parameters	Δρ_max_ = 0.31 e Å^−^^3^
0 restraints	Δρ_min_ = −0.24 e Å^−^^3^
Primary atom site location: structure-invariant direct methods	

### Special details

**Table:**

Geometry. All e.s.d.'s (except the e.s.d. in the dihedral angle between two l.s. planes) are estimated using the full covariance matrix. The cell e.s.d.'s are taken into account individually in the estimation of e.s.d.'s in distances, angles and torsion angles; correlations between e.s.d.'s in cell parameters are only used when they are defined by crystal symmetry. An approximate (isotropic) treatment of cell e.s.d.'s is used for estimating e.s.d.'s involving l.s. planes.
Refinement. Refinement was performed using all reflections. The weighted *R*-factor (*wR*) and goodness of fit (*S*) are based on *F*^2^. *R*-factor (gt) are based on *F*. The threshold expression of *F*^2^ > 2.0 σ(*F*^2^) is used only for calculating *R*-factor (gt).

### Fractional atomic coordinates and isotropic or equivalent isotropic displacement parameters (Å^2^)

**Table:**

	*x*	*y*	*z*	*U*_iso_*/*U*_eq_	
O1	0.45785 (19)	0.22041 (16)	0.07963 (5)	0.0513 (4)	
O2	0.3744 (3)	0.49657 (15)	0.22975 (5)	0.0564 (5)	
O3	0.4192 (2)	0.24905 (17)	0.28520 (5)	0.0529 (5)	
O4	0.5653 (3)	−0.00381 (17)	0.13489 (6)	0.0624 (5)	
O5	−0.1568 (3)	0.3544 (2)	0.09158 (9)	0.0951 (8)	
O6	−0.1984 (3)	0.3325 (3)	0.01141 (8)	0.0992 (8)	
N28	−0.1634 (3)	0.2779 (3)	0.05255 (8)	0.0639 (6)	
C7	0.4594 (3)	0.1130 (2)	0.20880 (7)	0.0379 (5)	
C8	0.5667 (3)	0.0171 (2)	0.18386 (8)	0.0453 (5)	
C9	0.6980 (3)	−0.0716 (3)	0.21168 (10)	0.0634 (7)	
C10	0.6446 (4)	−0.1116 (3)	0.26478 (10)	0.0688 (8)	
C11	0.5914 (4)	0.0305 (3)	0.29310 (9)	0.0646 (8)	
C12	0.4847 (3)	0.1371 (3)	0.26209 (8)	0.0446 (5)	
C13	0.3883 (3)	0.4839 (3)	0.18035 (8)	0.0425 (5)	
C14	0.4224 (4)	0.6324 (3)	0.15382 (8)	0.0550 (6)	
C15	0.3716 (4)	0.6286 (3)	0.09916 (9)	0.0633 (7)	
C16	0.4457 (4)	0.4907 (3)	0.07317 (9)	0.0582 (7)	
C17	0.4256 (3)	0.3441 (3)	0.10290 (8)	0.0430 (5)	
C18	0.3761 (3)	0.3469 (2)	0.15437 (7)	0.0361 (5)	
C19	0.3217 (3)	0.2019 (2)	0.18202 (7)	0.0356 (5)	
C20	0.2077 (3)	0.0996 (3)	0.15054 (8)	0.0429 (5)	
C21	0.0903 (3)	0.1434 (3)	0.12109 (10)	0.0617 (7)	
C22	−0.0262 (3)	0.0497 (3)	0.09109 (7)	0.0446 (5)	
C23	−0.0204 (3)	−0.1105 (3)	0.09304 (8)	0.0524 (6)	
C24	−0.1205 (4)	−0.2002 (3)	0.06219 (9)	0.0623 (7)	
C25	−0.2285 (4)	−0.1343 (3)	0.02791 (9)	0.0644 (7)	
C26	−0.2383 (3)	0.0213 (3)	0.02454 (8)	0.0585 (7)	
C27	−0.1403 (3)	0.1105 (3)	0.05629 (7)	0.0460 (5)	
H2	0.3781	0.4112	0.2428	0.0677*	
H4	0.5166	0.0675	0.1209	0.0748*	
H9A	0.7988	−0.0112	0.2135	0.0760*	
H9B	0.7217	−0.1649	0.1931	0.0760*	
H10A	0.5532	−0.1837	0.2630	0.0825*	
H10B	0.7356	−0.1603	0.2831	0.0825*	
H11A	0.5312	−0.0003	0.3231	0.0775*	
H11B	0.6895	0.0856	0.3045	0.0775*	
H14A	0.3632	0.7142	0.1708	0.0660*	
H14B	0.5398	0.6548	0.1564	0.0660*	
H15A	0.4083	0.7215	0.0825	0.0759*	
H15B	0.2518	0.6241	0.0964	0.0759*	
H16A	0.3936	0.4785	0.0399	0.0699*	
H16B	0.5627	0.5091	0.0680	0.0699*	
H19	0.2523	0.2392	0.2097	0.0427*	
H20	0.221 (6)	−0.001 (4)	0.1592 (14)	0.134 (14)*	
H21	0.083 (5)	0.251 (5)	0.1152 (14)	0.134 (14)*	
H23	0.0529	−0.1577	0.1157	0.0629*	
H24	−0.1145	−0.3064	0.0647	0.0747*	
H25	−0.2949	−0.1956	0.0070	0.0773*	
H26	−0.3102	0.0670	0.0011	0.0701*	

### Atomic displacement parameters (Å^2^)

**Table:**

	*U*^11^	*U*^22^	*U*^33^	*U*^12^	*U*^13^	*U*^23^
O1	0.0567 (10)	0.0544 (9)	0.0427 (8)	0.0037 (7)	0.0023 (7)	−0.0073 (7)
O2	0.0823 (13)	0.0447 (8)	0.0421 (8)	0.0114 (8)	−0.0029 (8)	−0.0048 (7)
O3	0.0658 (11)	0.0523 (9)	0.0403 (8)	0.0022 (8)	−0.0093 (7)	−0.0046 (7)
O4	0.0779 (13)	0.0533 (10)	0.0561 (10)	0.0166 (8)	0.0095 (9)	−0.0027 (8)
O5	0.1043 (18)	0.0674 (12)	0.1119 (17)	0.0224 (11)	−0.0542 (14)	−0.0214 (12)
O6	0.1102 (18)	0.1062 (16)	0.0808 (13)	0.0340 (14)	−0.0112 (13)	0.0364 (12)
N28	0.0511 (13)	0.0701 (13)	0.0699 (13)	0.0104 (10)	−0.0181 (10)	0.0060 (11)
C7	0.0336 (11)	0.0372 (10)	0.0425 (11)	−0.0030 (8)	−0.0069 (9)	0.0000 (8)
C8	0.0402 (12)	0.0375 (10)	0.0581 (13)	−0.0008 (9)	−0.0023 (10)	0.0005 (10)
C9	0.0483 (15)	0.0515 (13)	0.0900 (18)	0.0082 (11)	−0.0104 (13)	0.0025 (13)
C10	0.0639 (18)	0.0549 (14)	0.0867 (18)	0.0059 (12)	−0.0259 (14)	0.0135 (13)
C11	0.0783 (19)	0.0516 (13)	0.0629 (15)	−0.0011 (12)	−0.0363 (14)	0.0062 (11)
C12	0.0450 (13)	0.0394 (11)	0.0490 (12)	−0.0074 (9)	−0.0129 (10)	0.0012 (9)
C13	0.0407 (12)	0.0432 (11)	0.0435 (11)	0.0072 (9)	−0.0064 (9)	−0.0015 (9)
C14	0.0628 (16)	0.0409 (11)	0.0612 (14)	0.0018 (10)	−0.0029 (12)	0.0036 (10)
C15	0.0778 (19)	0.0517 (13)	0.0602 (14)	0.0097 (12)	−0.0019 (13)	0.0142 (11)
C16	0.0691 (17)	0.0585 (14)	0.0473 (12)	0.0079 (12)	0.0064 (11)	0.0138 (11)
C17	0.0387 (12)	0.0479 (11)	0.0422 (11)	0.0014 (9)	−0.0057 (9)	0.0026 (9)
C18	0.0312 (11)	0.0406 (10)	0.0365 (10)	0.0015 (8)	−0.0053 (8)	0.0014 (8)
C19	0.0315 (11)	0.0392 (10)	0.0360 (9)	0.0003 (8)	−0.0034 (8)	−0.0012 (8)
C20	0.0363 (12)	0.0446 (11)	0.0474 (11)	−0.0035 (9)	−0.0084 (9)	0.0029 (10)
C21	0.0515 (16)	0.0474 (13)	0.0852 (18)	0.0090 (11)	−0.0331 (13)	−0.0134 (12)
C22	0.0351 (12)	0.0516 (12)	0.0468 (11)	0.0007 (9)	−0.0072 (9)	−0.0051 (10)
C23	0.0493 (14)	0.0511 (12)	0.0564 (13)	0.0025 (10)	−0.0149 (11)	−0.0054 (10)
C24	0.0629 (17)	0.0584 (14)	0.0655 (15)	−0.0078 (12)	−0.0021 (12)	−0.0161 (12)
C25	0.0573 (17)	0.0815 (18)	0.0543 (14)	−0.0151 (13)	−0.0092 (12)	−0.0254 (13)
C26	0.0436 (14)	0.0909 (18)	0.0406 (12)	−0.0009 (12)	−0.0105 (10)	−0.0045 (12)
C27	0.0378 (12)	0.0574 (13)	0.0424 (11)	0.0030 (9)	−0.0059 (9)	−0.0007 (10)

### Geometric parameters (Å, °)

**Table:**

O1—C17	1.269 (3)	C23—C24	1.380 (4)
O2—C13	1.308 (3)	C24—C25	1.371 (4)
O3—C12	1.270 (3)	C25—C26	1.363 (4)
O4—C8	1.299 (3)	C26—C27	1.381 (4)
O5—N28	1.224 (3)	O2—H2	0.820
O6—N28	1.210 (3)	O4—H4	0.820
N28—C27	1.476 (3)	C9—H9A	0.970
C7—C8	1.377 (3)	C9—H9B	0.970
C7—C12	1.427 (3)	C10—H10A	0.970
C7—C19	1.520 (3)	C10—H10B	0.970
C8—C9	1.495 (4)	C11—H11A	0.970
C9—C10	1.507 (4)	C11—H11B	0.970
C10—C11	1.512 (4)	C14—H14A	0.970
C11—C12	1.500 (4)	C14—H14B	0.970
C13—C14	1.499 (3)	C15—H15A	0.970
C13—C18	1.379 (3)	C15—H15B	0.970
C14—C15	1.488 (4)	C16—H16A	0.970
C15—C16	1.511 (4)	C16—H16B	0.970
C16—C17	1.509 (3)	C19—H19	0.980
C17—C18	1.415 (3)	C20—H20	0.91 (4)
C18—C19	1.527 (3)	C21—H21	0.96 (4)
C19—C20	1.519 (3)	C23—H23	0.930
C20—C21	1.273 (4)	C24—H24	0.930
C21—C22	1.468 (4)	C25—H25	0.930
C22—C23	1.400 (3)	C26—H26	0.930
C22—C27	1.393 (3)		
			
O1···O4	2.579 (2)	O2···H20^iii^	3.03 (4)
O1···C7	3.519 (3)	O3···H9A^v^	3.0932
O1···C8	3.368 (3)	O3···H9B^v^	3.0427
O1···C13	3.555 (3)	O3···H10B^v^	3.4297
O1···C19	2.924 (3)	O3···H14A^iv^	2.5908
O1···C20	2.962 (3)	O3···H15B^iv^	3.5910
O1···C21	3.247 (3)	O3···H20^iii^	2.87 (4)
O2···O3	2.627 (2)	O3···H23^iii^	2.7324
O2···C7	3.462 (3)	O4···H14A^vi^	3.1070
O2···C12	3.367 (3)	O4···H14B^vi^	3.0395
O2···C19	2.889 (3)	O4···H15A^vi^	3.0330
O3···C8	3.563 (3)	O5···H10B^iii^	3.3645
O3···C13	3.439 (3)	O5···H11A^iii^	2.7557
O3···C18	3.553 (3)	O5···H11B^iii^	3.4070
O3···C19	2.841 (3)	O5···H16B^vii^	2.7012
O4···C10	3.587 (3)	O5···H24^x^	3.0638
O4···C17	3.342 (3)	O6···H15A^viii^	3.0095
O4···C18	3.462 (3)	O6···H15B^viii^	2.8817
O4···C19	2.949 (3)	O6···H16A^viii^	2.6382
O4···C20	3.060 (3)	O6···H16B^vii^	2.8973
O5···C21	2.816 (4)	O6···H24^x^	3.5097
O5···C22	2.860 (3)	O6···H24^ii^	3.2529
O5···C26	3.456 (4)	N28···H16B^vii^	3.0259
O6···C22	3.510 (3)	C7···H2^iv^	3.5002
O6···C26	2.757 (4)	C7···H10B^v^	3.1650
N28···C21	2.952 (4)	C8···H10B^v^	3.3457
C7···C10	2.860 (4)	C8···H14A^vi^	3.1288
C7···C13	3.369 (3)	C8···H14B^vi^	3.2486
C7···C17	3.443 (3)	C9···H11B^ix^	3.1575
C8···C11	2.875 (4)	C9···H14A^vi^	3.4471
C8···C18	3.349 (3)	C9···H14B^vi^	3.0638
C8···C20	3.102 (3)	C10···H9A^ix^	3.5614
C9···C12	2.847 (4)	C10···H11B^ix^	3.4871
C12···C18	3.472 (3)	C10···H14B^vi^	3.5913
C13···C16	2.860 (4)	C10···H19^iv^	3.5334
C14···C17	2.849 (3)	C11···H9B^v^	3.0754
C15···C18	2.854 (3)	C11···H14B^ix^	3.4199
C17···C20	3.047 (3)	C12···H2^iv^	3.5325
C17···C21	3.266 (4)	C12···H9A^v^	3.5861
C18···C21	3.032 (3)	C12···H9B^v^	3.1492
C20···C23	2.992 (3)	C12···H10B^v^	3.1173
C22···C25	2.815 (4)	C12···H14A^iv^	3.4067
C23···C26	2.751 (4)	C13···H10B^v^	3.4141
C24···C27	2.720 (4)	C13···H11A^iii^	3.3914
O1···O5^i^	3.335 (3)	C13···H11B^v^	3.5415
O1···O6^i^	3.467 (3)	C14···H9B^x^	3.1573
O1···N28^i^	3.191 (3)	C14···H10A^x^	3.4394
O1···C25^ii^	3.438 (3)	C14···H11B^v^	3.3315
O1···C26^i^	3.353 (3)	C14···H20^x^	3.60 (4)
O1···C27^i^	3.449 (3)	C15···H23^x^	3.2135
O2···O3^iii^	3.256 (3)	C16···H16A^xi^	3.2693
O2···C7^iii^	3.321 (3)	C16···H25^ii^	3.5326
O2···C12^iii^	3.159 (3)	C17···H25^ii^	3.3230
O2···C19^iii^	3.346 (3)	C18···H10B^v^	3.5206
O2···C20^iii^	3.345 (3)	C19···H10A^iii^	3.5190
O3···O2^iv^	3.256 (3)	C20···H2^iv^	3.3300
O3···C9^v^	3.466 (3)	C21···H11A^iii^	3.5807
O3···C13^iv^	3.522 (3)	C22···H9A^vii^	3.5700
O3···C14^iv^	3.368 (3)	C23···H9B^vii^	3.4100
O3···C23^iii^	3.510 (3)	C23···H15B^vi^	3.1937
O4···C14^vi^	3.417 (3)	C24···H15B^vi^	3.4815
O4···C22^i^	3.541 (3)	C25···H15A^xii^	3.5163
O4···C25^i^	3.478 (3)	C26···H4^vii^	3.2613
O4···C26^i^	3.331 (3)	C27···H4^vii^	3.2928
O4···C27^i^	3.325 (3)	H2···C7^iii^	3.5002
O5···O1^vii^	3.335 (3)	H2···C12^iii^	3.5325
O5···C11^iii^	3.430 (4)	H2···C20^iii^	3.3300
O5···C16^vii^	3.452 (4)	H2···H9A^v^	2.9166
O5···C17^vii^	3.392 (4)	H2···H10A^iii^	3.5826
O6···O1^vii^	3.467 (3)	H2···H10B^v^	3.2636
O6···C15^viii^	3.223 (4)	H2···H14A^iv^	3.4703
O6···C16^vii^	3.597 (4)	H2···H19^iii^	3.3010
O6···C16^viii^	3.343 (4)	H2···H20^iii^	2.8135
O6···C24^ii^	3.442 (4)	H4···C26^i^	3.2613
N28···O1^vii^	3.191 (3)	H4···C27^i^	3.2928
C7···O2^iv^	3.321 (3)	H4···H14A^vi^	3.5776
C9···O3^ix^	3.466 (3)	H4···H15A^vi^	3.2979
C11···O5^iv^	3.430 (4)	H4···H26^i^	3.4624
C12···O2^iv^	3.159 (3)	H9A···O2^ix^	3.0144
C13···O3^iii^	3.522 (3)	H9A···O3^ix^	3.0932
C14···O3^iii^	3.368 (3)	H9A···C10^v^	3.5614
C14···O4^x^	3.417 (3)	H9A···C12^ix^	3.5861
C15···O6^viii^	3.223 (4)	H9A···C22^i^	3.5700
C16···O5^i^	3.452 (4)	H9A···H2^ix^	2.9166
C16···O6^i^	3.597 (4)	H9A···H10A^v^	3.1552
C16···O6^viii^	3.343 (4)	H9A···H10B^v^	3.0761
C17···O5^i^	3.392 (4)	H9A···H11B^ix^	3.5519
C19···O2^iv^	3.346 (3)	H9A···H23^i^	3.5489
C20···O2^iv^	3.345 (3)	H9B···O3^ix^	3.0427
C22···O4^vii^	3.541 (3)	H9B···C11^ix^	3.0754
C23···O3^iv^	3.510 (3)	H9B···C12^ix^	3.1492
C24···O6^ii^	3.442 (4)	H9B···C14^vi^	3.1573
C25···O1^ii^	3.438 (3)	H9B···C23^i^	3.4100
C25···O4^vii^	3.478 (3)	H9B···H11A^ix^	3.5713
C26···O1^vii^	3.353 (3)	H9B···H11B^ix^	2.2934
C26···O4^vii^	3.331 (3)	H9B···H14A^vi^	3.1297
C27···O1^vii^	3.449 (3)	H9B···H14B^vi^	2.3497
C27···O4^vii^	3.325 (3)	H9B···H23^i^	3.3834
O1···H4	1.7797	H10A···O2^vi^	3.2557
O1···H16A	2.5328	H10A···C14^vi^	3.4394
O1···H16B	2.6764	H10A···C19^iv^	3.5190
O1···H20	3.45 (4)	H10A···H2^iv^	3.5826
O1···H21	3.20 (4)	H10A···H9A^ix^	3.1552
O2···H14A	2.4515	H10A···H11B^ix^	3.4095
O2···H14B	2.7342	H10A···H14A^vi^	2.9857
O2···H19	2.5059	H10A···H14B^vi^	3.1345
O3···H2	1.8284	H10A···H19^iv^	2.6664
O3···H11A	2.5527	H10A···H21^iv^	3.4421
O3···H11B	2.6512	H10B···O2^ix^	3.4565
O3···H19	2.3833	H10B···O3^ix^	3.4297
O4···H9A	2.7765	H10B···O5^iv^	3.3645
O4···H9B	2.4191	H10B···C7^ix^	3.1650
O4···H20	2.86 (5)	H10B···C8^ix^	3.3457
O5···H21	2.21 (4)	H10B···C12^ix^	3.1173
O6···H21	3.59 (4)	H10B···C13^ix^	3.4141
O6···H26	2.4996	H10B···C18^ix^	3.5206
N28···H21	2.57 (4)	H10B···H2^ix^	3.2636
N28···H26	2.5635	H10B···H9A^ix^	3.0761
C7···H2	2.8310	H10B···H11B^ix^	3.2578
C7···H4	2.3932	H11A···O2^iv^	3.5398
C7···H9A	2.9491	H11A···O5^iv^	2.7557
C7···H9B	3.2507	H11A···C13^iv^	3.3914
C7···H10A	3.0455	H11A···C21^iv^	3.5807
C7···H11A	3.2052	H11A···H9B^v^	3.5713
C7···H11B	3.1136	H11A···H15B^iv^	3.3195
C7···H20	2.51 (4)	H11A···H19^iv^	3.3316
C8···H10A	2.7220	H11A···H21^iv^	2.8645
C8···H10B	3.3077	H11A···H23^iii^	3.4653
C8···H11B	3.3598	H11A···H24^iii^	3.4561
C8···H19	3.2735	H11B···O5^iv^	3.4070
C8···H20	2.86 (5)	H11B···C9^v^	3.1575
C9···H4	3.0330	H11B···C10^v^	3.4871
C9···H11A	3.2983	H11B···C13^ix^	3.5415
C9···H11B	2.7984	H11B···C14^ix^	3.3315
C11···H9A	2.7222	H11B···H9A^v^	3.5519
C11···H9B	3.3157	H11B···H9B^v^	2.2934
C12···H2	2.5899	H11B···H10A^v^	3.4095
C12···H9A	3.1361	H11B···H10B^v^	3.2578
C12···H10A	2.8535	H11B···H14B^ix^	2.4774
C12···H10B	3.3335	H14A···O3^iii^	2.5908
C12···H19	2.4772	H14A···O4^x^	3.1070
C13···H15A	3.3079	H14A···C8^x^	3.1288
C13···H15B	2.7399	H14A···C9^x^	3.4471
C13···H16B	3.2943	H14A···C12^iii^	3.4067
C13···H19	2.5268	H14A···H2^iii^	3.4703
C14···H2	3.0552	H14A···H4^x^	3.5776
C14···H16A	3.2842	H14A···H9B^x^	3.1297
C14···H16B	2.7552	H14A···H10A^x^	2.9857
C16···H14A	3.2958	H14A···H19^iii^	3.2957
C16···H14B	2.7130	H14A···H20^x^	2.7563
C17···H4	2.5654	H14A···H23^x^	3.0853
C17···H14B	3.1843	H14B···O4^x^	3.0395
C17···H15A	3.3387	H14B···C8^x^	3.2486
C17···H15B	2.8219	H14B···C9^x^	3.0638
C17···H19	3.2829	H14B···C10^x^	3.5913
C17···H21	2.91 (4)	H14B···C11^v^	3.4199
C18···H2	2.3882	H14B···H9B^x^	2.3497
C18···H4	2.8343	H14B···H10A^x^	3.1345
C18···H14A	3.2352	H14B···H11B^v^	2.4774
C18···H14B	2.9946	H15A···O4^x^	3.0330
C18···H15B	3.0226	H15A···O6^viii^	3.0095
C18···H16A	3.2228	H15A···C25^xiii^	3.5163
C18···H16B	3.0842	H15A···H4^x^	3.2979
C18···H20	3.28 (4)	H15A···H20^x^	3.5105
C18···H21	2.70 (4)	H15A···H23^x^	3.1925
C19···H2	2.4632	H15A···H25^xiii^	3.2146
C19···H4	2.5495	H15A···H26^viii^	2.9669
C19···H21	2.63 (4)	H15B···O3^iii^	3.5910
C20···H4	2.6397	H15B···O6^viii^	2.8817
C20···H23	2.7218	H15B···C23^x^	3.1937
C21···H4	3.5068	H15B···C24^x^	3.4815
C21···H19	2.7814	H15B···H11A^iii^	3.3195
C21···H23	2.6486	H15B···H23^x^	2.5472
C22···H20	2.70 (4)	H15B···H24^x^	3.1222
C22···H24	3.2604	H16A···O6^viii^	2.6382
C22···H26	3.2731	H16A···C16^xi^	3.2693
C23···H20	2.76 (4)	H16A···H16A^xi^	2.7561
C23···H21	3.32 (4)	H16A···H16B^xi^	2.8642
C23···H25	3.2288	H16A···H25^ii^	2.8676
C24···H26	3.2078	H16B···O5^i^	2.7012
C25···H23	3.2212	H16B···O6^i^	2.8973
C26···H24	3.2027	H16B···N28^i^	3.0259
C27···H21	2.66 (4)	H16B···H16A^xi^	2.8642
C27···H23	3.2058	H16B···H24^xiii^	3.0668
C27···H25	3.2123	H16B···H25^xiii^	3.2534
H2···H14A	3.2518	H19···O2^iv^	2.8468
H2···H14B	3.3843	H19···C10^iii^	3.5334
H2···H19	2.0038	H19···H2^iv^	3.3010
H4···H9A	3.3788	H19···H10A^iii^	2.6664
H4···H9B	3.2183	H19···H11A^iii^	3.3316
H4···H19	3.5186	H19···H14A^iv^	3.2957
H4···H20	2.6720	H20···O2^iv^	3.03 (4)
H9A···H10A	2.8225	H20···O3^iv^	2.87 (4)
H9A···H10B	2.3061	H20···C14^vi^	3.60 (4)
H9A···H11B	2.6954	H20···H2^iv^	2.8135
H9B···H10A	2.3074	H20···H14A^vi^	2.7563
H9B···H10B	2.3641	H20···H15A^vi^	3.5105
H10A···H11A	2.2564	H21···H10A^iii^	3.4421
H10A···H11B	2.8078	H21···H11A^iii^	2.8645
H10B···H11A	2.4148	H23···O3^iv^	2.7324
H10B···H11B	2.2502	H23···C15^vi^	3.2135
H14A···H15A	2.3527	H23···H9A^vii^	3.5489
H14A···H15B	2.2789	H23···H9B^vii^	3.3834
H14B···H15A	2.2751	H23···H11A^iv^	3.4653
H14B···H15B	2.8045	H23···H14A^vi^	3.0853
H14B···H16B	2.6552	H23···H15A^vi^	3.1925
H15A···H16A	2.3988	H23···H15B^vi^	2.5472
H15A···H16B	2.2686	H24···O5^vi^	3.0638
H15B···H16A	2.2743	H24···O6^vi^	3.5097
H15B···H16B	2.8148	H24···O6^ii^	3.2529
H15B···H21	3.5649	H24···H11A^iv^	3.4561
H19···H20	2.4879	H24···H15B^vi^	3.1222
H19···H21	2.8185	H24···H16B^xii^	3.0668
H20···H21	2.72 (6)	H25···O1^ii^	2.6218
H20···H23	2.2321	H25···C16^ii^	3.5326
H21···H23	3.5786	H25···C17^ii^	3.3230
H23···H24	2.2928	H25···H15A^xii^	3.2146
H24···H25	2.2988	H25···H16A^ii^	2.8676
H25···H26	2.3001	H25···H16B^xii^	3.2534
O1···H25^ii^	2.6218	H25···H26^xiv^	3.3855
O1···H26^i^	3.1092	H26···O1^vii^	3.1092
O1···H26^ii^	3.4836	H26···O1^ii^	3.4836
O2···H9A^v^	3.0144	H26···H4^vii^	3.4624
O2···H10A^x^	3.2557	H26···H15A^viii^	2.9669
O2···H10B^v^	3.4565	H26···H25^xiv^	3.3855
O2···H11A^iii^	3.5398	H26···H26^xiv^	3.2819
O2···H19^iii^	2.8468		
			
O5—N28—O6	122.7 (3)	C8—C9—H9B	109.396
O5—N28—C27	118.7 (2)	C10—C9—H9A	109.398
O6—N28—C27	118.5 (2)	C10—C9—H9B	109.398
C8—C7—C12	118.27 (18)	H9A—C9—H9B	108.010
C8—C7—C19	123.45 (17)	C9—C10—H10A	109.530
C12—C7—C19	118.18 (17)	C9—C10—H10B	109.527
O4—C8—C7	124.04 (19)	C11—C10—H10A	109.524
O4—C8—C9	114.01 (19)	C11—C10—H10B	109.522
C7—C8—C9	121.9 (2)	H10A—C10—H10B	108.089
C8—C9—C10	111.2 (2)	C10—C11—H11A	108.754
C9—C10—C11	110.6 (2)	C10—C11—H11B	108.754
C10—C11—C12	114.0 (2)	C12—C11—H11A	108.752
O3—C12—C7	121.82 (18)	C12—C11—H11B	108.748
O3—C12—C11	117.23 (19)	H11A—C11—H11B	107.664
C7—C12—C11	120.94 (18)	C13—C14—H14A	109.140
O2—C13—C14	114.00 (17)	C13—C14—H14B	109.137
O2—C13—C18	123.87 (18)	C15—C14—H14A	109.150
C14—C13—C18	122.12 (19)	C15—C14—H14B	109.159
C13—C14—C15	112.29 (19)	H14A—C14—H14B	107.867
C14—C15—C16	110.4 (2)	C14—C15—H15A	109.576
C15—C16—C17	113.3 (2)	C14—C15—H15B	109.565
O1—C17—C16	116.59 (19)	C16—C15—H15A	109.587
O1—C17—C18	122.50 (18)	C16—C15—H15B	109.585
C16—C17—C18	120.91 (18)	H15A—C15—H15B	108.120
C13—C18—C17	117.93 (18)	C15—C16—H16A	108.917
C13—C18—C19	120.13 (17)	C15—C16—H16B	108.926
C17—C18—C19	121.81 (16)	C17—C16—H16A	108.930
C7—C19—C18	115.44 (16)	C17—C16—H16B	108.927
C7—C19—C20	112.70 (15)	H16A—C16—H16B	107.735
C18—C19—C20	113.87 (16)	C7—C19—H19	104.427
C19—C20—C21	126.5 (2)	C18—C19—H19	104.434
C20—C21—C22	128.7 (2)	C20—C19—H19	104.428
C21—C22—C23	121.05 (19)	C19—C20—H20	111 (3)
C21—C22—C27	123.57 (19)	C21—C20—H20	122 (3)
C23—C22—C27	115.21 (19)	C20—C21—H21	116 (3)
C22—C23—C24	121.7 (2)	C22—C21—H21	115 (3)
C23—C24—C25	120.6 (3)	C22—C23—H23	119.134
C24—C25—C26	119.7 (3)	C24—C23—H23	119.136
C25—C26—C27	119.3 (3)	C23—C24—H24	119.669
N28—C27—C22	120.16 (19)	C25—C24—H24	119.681
N28—C27—C26	116.5 (2)	C24—C25—H25	120.135
C22—C27—C26	123.3 (2)	C26—C25—H25	120.133
C13—O2—H2	109.476	C25—C26—H26	120.338
C8—O4—H4	109.473	C27—C26—H26	120.342
C8—C9—H9A	109.394		
			
O5—N28—C27—C22	−37.3 (3)	C13—C14—C15—C16	−52.0 (3)
O5—N28—C27—C26	141.7 (2)	C14—C15—C16—C17	47.0 (3)
O6—N28—C27—C22	147.3 (2)	C15—C16—C17—O1	167.85 (19)
O6—N28—C27—C26	−33.7 (3)	C15—C16—C17—C18	−13.3 (3)
C8—C7—C12—O3	162.73 (18)	O1—C17—C18—C13	162.33 (17)
C8—C7—C12—C11	−16.9 (3)	O1—C17—C18—C19	−13.6 (3)
C12—C7—C8—O4	−173.91 (17)	C16—C17—C18—C13	−16.5 (3)
C12—C7—C8—C9	5.3 (3)	C16—C17—C18—C19	167.59 (18)
C8—C7—C19—C18	−78.8 (3)	C13—C18—C19—C7	−86.4 (2)
C8—C7—C19—C20	54.4 (3)	C13—C18—C19—C20	140.88 (17)
C19—C7—C8—O4	2.3 (3)	C17—C18—C19—C7	89.4 (2)
C19—C7—C8—C9	−178.46 (15)	C17—C18—C19—C20	−43.3 (3)
C12—C7—C19—C18	97.47 (19)	C7—C19—C20—C21	−174.18 (18)
C12—C7—C19—C20	−129.31 (17)	C18—C19—C20—C21	−40.2 (3)
C19—C7—C12—O3	−13.7 (3)	C19—C20—C21—C22	−178.03 (19)
C19—C7—C12—C11	166.64 (15)	C20—C21—C22—C23	1.3 (4)
O4—C8—C9—C10	−150.09 (17)	C20—C21—C22—C27	−173.7 (3)
C7—C8—C9—C10	30.6 (3)	C21—C22—C23—C24	−174.94 (19)
C8—C9—C10—C11	−53.8 (3)	C21—C22—C27—N28	−7.7 (3)
C9—C10—C11—C12	43.6 (3)	C21—C22—C27—C26	173.38 (18)
C10—C11—C12—O3	171.66 (19)	C23—C22—C27—N28	177.06 (17)
C10—C11—C12—C7	−8.7 (3)	C23—C22—C27—C26	−1.9 (3)
O2—C13—C14—C15	−156.97 (18)	C27—C22—C23—C24	0.4 (3)
O2—C13—C18—C17	−167.73 (18)	C22—C23—C24—C25	0.7 (4)
O2—C13—C18—C19	8.3 (3)	C23—C24—C25—C26	−0.5 (4)
C14—C13—C18—C17	11.0 (3)	C24—C25—C26—C27	−0.8 (4)
C14—C13—C18—C19	−172.96 (18)	C25—C26—C27—N28	−176.85 (19)
C18—C13—C14—C15	24.2 (3)	C25—C26—C27—C22	2.1 (4)

Symmetry codes: (i) *x*+1, *y*, *z*; (ii) −*x*, −*y*, −*z*; (iii) −*x*+1/2, *y*+1/2, −*z*+1/2; (iv) −*x*+1/2, *y*−1/2, −*z*+1/2; (v) −*x*+3/2, *y*+1/2, −*z*+1/2; (vi) *x*, *y*−1, *z*; (vii) *x*−1, *y*, *z*; (viii) −*x*, −*y*+1, −*z*; (ix) −*x*+3/2, *y*−1/2, −*z*+1/2; (x) *x*, *y*+1, *z*; (xi) −*x*+1, −*y*+1, −*z*; (xii) *x*−1, *y*−1, *z*; (xiii) *x*+1, *y*+1, *z*; (xiv) −*x*−1, −*y*, −*z*.

### Hydrogen-bond geometry (Å, °)

**Table:**

*D*—H···*A*	*D*—H	H···*A*	*D*···*A*	*D*—H···*A*
O2—H2···O3	0.82	1.83	2.627 (2)	164.
O4—H4···O1	0.82	1.78	2.579 (2)	165.
